# Vulnerability-Based Spatial Sampling Stratification for the National Children’s Study, Worcester County, Massachusetts: Capturing Health-Relevant Environmental and Sociodemographic Variability

**DOI:** 10.1289/ehp.0901315

**Published:** 2010-03-08

**Authors:** Timothy J. Downs, Yelena Ogneva-Himmelberger, Onesky Aupont, Yangyang Wang, Ann Raj, Paula Zimmerman, Robert Goble, Octavia Taylor, Linda Churchill, Celeste Lemay, Thomas McLaughlin, Marianne Felice

**Affiliations:** 1 Environmental Science and Policy Program; 2 George Perkins Marsh Research Institute and; 3 Geographic Information Science for Development and Environment Program, Clark University, Worcester, Massachusetts, USA; 4 Department of Pediatrics, University of Massachusetts Medical School, Worcester, Massachusetts, USA; 5 School of Geography, Clark University, Worcester, Massachusetts, USA

**Keywords:** environmental health, GIS, National Children’s Study, stratified sampling, vulnerability

## Abstract

**Background:**

The National Children’s Study is the most ambitious study ever attempted in the United States to assess how environmental factors impact child health and development. It aims to follow 100,000 children from gestation until 21 years of age. Success requires breaking new interdisciplinary ground, starting with how to select the sample of > 1,000 children in each of 105 study sites; no standardized protocol exists for stratification of the target population by factoring in the diverse environments it inhabits. Worcester County, Massachusetts, like other sites, stratifies according to local conditions and local knowledge, subject to probability sampling rules.

**Objectives:**

We answer the following questions: How do we divide Worcester County into viable strata that represent its health-relevant environmental and sociodemographic heterogeneity, subject to sampling rules? What potential does our approach have to inform stratification at other sites?

**Results:**

We developed a multivariable, vulnerability-based method for spatial sampling consisting of two descriptive indices: a hazards/stressors exposure index (comprising three proxy variables), and an adaptive capacity/sociodemographic character index (five variables). Multivariable, health-relevant stratification at the start of the study may improve detection power for environment–child health associations down the line. Eighteen strata capture countywide heterogeneity in the indices and have optimal relative homogeneity within each. They achieve comparable expected birth counts and conform to local concepts of space.

**Conclusion:**

The approach offers moderate to high potential to inform other sites, limited by intersite differences in data availability, geodemographics, and technical capacity. Energetic community engagement from the start promotes local stratification coherence, plus vital researcher–community trust and co-ownership for sustainability.

The National Children’s Study (NCS) is the most ambitious study ever attempted in the United States, and arguably the world, to assess how environmental factors affect child health and development. To complete the project, 100,000 children from across the United States—the largest pregnancy cohort in the history of the country—will be followed from gestation until 21 years of age. The ultimate goal of the study is to improve the health and well-being of children (NCS 2010). Its appropriately broad definition of environment encompasses the following:

- Natural and human-made environmental factors
- Biological and chemical factors
- Physical surroundings
- Social factors
- Cultural and family influences and differences
- Geographic locations.

Population health disparities are viewed as a function of gradients in environmental exposures; health care access, utilization, or quality; and health status (Carter-Pokras and Baquet 2002; Gibbons 2005). The NCS will combine direct measures of environmental media and biological specimens with indirect questionnaire measures (e.g., health care access, utilization, and quality; health status, age, sex, income, education) and extant demographic, geographic, and environmental data (Needham et al. 2005). The NCS represents a strategic opportunity to synthesize lessons and methods from epidemiology, clinical science, and risk assessment (Gilliland et al. 2005; Trasande et al. 2009).

To reach NCS goals, new interdisciplinary methods are needed, including methods to select a sample representative of children nationwide, a “unique and demanding challenge” (Scheidt et al. 2009). As a first sampling stage, NCS investigators chose 105 study sites based on geographic and demographic characteristics, including 10 pilot Vanguard Centers and 95 other sites to be implemented in three waves (waves 1, 2, and 3). Investigators must then determine site-specific protocols to stratify local populations in accordance with health-relevant social and environmental characteristics, to maximize their power to detect associations. For example, one Vanguard Center, Queens County, New York, used community boundaries previously defined in 2007 by the New York City Department of City Planning to delineate 18 strata, and then checked for homogeneity within strata using census data on race, ethnicity, education, income, and foreign-born status (Lioy et al. 2009).

In this report we describe the stratification process developed for the wave 1 Worcester County, Massachusetts, site that began in 2007 as the Mass CHILD (Massachusetts Child Health Indicators and Life Determinants) project under the aegis of the University of Massachusetts Medical School. Specifically, we discuss the process used to divide Worcester County into viable strata that represent its health-relevant environmental and sociodemographic variability, and the potential our approach has to inform stratification elsewhere.

## Worcester County

**Figure 1:**
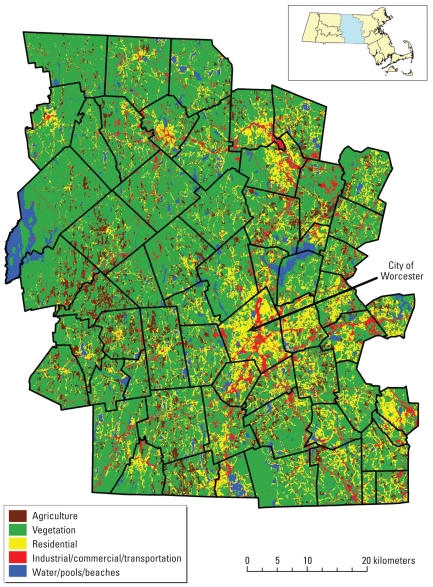
Map of Worcester County. Shows land use/land cover types, town boundaries and major roads. Data from MassGIS (2009).

Worcester County, Massachusetts, comprises 60 diverse towns, including the city of Worcester, the second-largest city (population 173,000, density 4,700 per mi^2^ in 2000) in New England (Figure 1). In the mid- to late 19th century, the city of Worcester and the Blackstone River Valley were the epicenter of the U.S. Industrial Revolution, a bustling place of canals, mills, and factories. This history, however, also means it suffers from an inherited, persistent pollution burden (e.g., lead in much of the soil, polychlorinated biphenyls in some pond/lake sediments). At the other end of the spectrum, the rural town of Petersham numbered a population of < 1,200 in 2000, yet is the second-largest land area (density only 22 per mi^2^ in 2000), comprising large tracts of woodland, conservation land, and scattered small farms.

## Materials and Methods

### Vulnerability theory

We used vulnerability theory to inform our sampling method because it contemplates both exposure to stressors and adaptive capacity, lending itself to the expansive notion of environment. Vulnerability to adverse health and developmental outcomes is also influenced by age, sex, and genetics. An extension of risk theory, vulnerability has been defined as “differential capacity to deal with hazards, based on the position of groups and individuals within both the physical and social worlds” [Ahmad et al. 2001; NEJAC (National Environmental Justice Advisory Council)/U.S. Environmental Protection Agency (EPA) 2004]. Dimensions of vulnerability include

- Differential exposure to risk agents or stressors (e.g., toxics, blight, crime—a function of the physical and social environments)
- Differential susceptibility and sensitivity to adverse consequences of exposure. Whereas susceptibility is predisposition or lack of resistance and is related to the likelihood of outcomes occurring, sensitivity is related to how that likelihood changes with changes in exposure; and both are functions of genetics, sex, age, and immune status.
- Differential individual and group coping/adaptive capacity and preparedness to respond—a function of biological variables such as health status and socioeconomic variables such as access to financial resources, sociopolitical networks, and information
- Differential individual and group resilience/ability to recover from adverse effects—also a function of sociobiological variables (Downs and Larson 2007).

### Sampling theory

The sampling frame for the NCS consists of a list of women who are pregnant or likely to become pregnant and who reside in the selected segments/strata at the time of sampling and recruitment (one-quarter of total sample size each year for 4 years, anticipated 2011/2012 to 2014/2015 for our site). The probability of selection is equal for all women in the sampling frame at all stages of the selection process. Eligible women are selected independently of one another. Spatial sampling is used to define segments/strata from which the probability sample is drawn. It starts with construction of a frame that separates eligible women into strata according to where they live. Strata must be relatively homogeneous within each stratum (small variances of attributes chosen), but heterogeneous among themselves (Mulvey 1983), representing the heterogeneity of vulnerability in the county. In addition, strata were limited to a reasonably small number (preferably 12–15 and not exceeding 20) and had to fully cover the county, be contiguous, have comparable (mean ± 10%) measures of size (MOS; expected births/year), and conform to local concepts of space to be coherent to local people. Each stratum is subsequently subdivided into smaller spatial segments with comparable MOS that also conform to local neighborhood characteristics. One segment is then randomly selected from each stratum, and together these segments constitute the representative random sample of geographic space in the county inhabited by women who are pregnant or likely to become pregnant (Montaquila et al. 2007).

During the stratification process, data at four spatial scales were considered: census block, census block-group, census tract, and town. We considered the census block-group to be the most appropriate scale because it is neither too small with too much data nor too large to lose spatial resolution. In addition, valuable sociodemographic data from U.S. Census are readily available at the block-group scale. We used Census 2000 data at the block-group level (U.S. Census Bureau 2001) as the basis for aggregation and used more recent intercensus data available for areas that have populations of 20,000 or more (currently, 6 of 60 towns in Worcester County, according to the 2008 population estimates from the U.S. Census Bureau’s web page, accessed 14 December 2009). There are 595 block-groups in Worcester County, Massachusetts. On average, 1,262 people were living in a census block-group in 2000, but with wide variation (SD 650; range 104–4,332).

### Descriptive vulnerability indices

We created two spatially explicit descriptive indices, one comprising proxies for hazards and stressors exposure (H), the second comprising indicators that describe adaptive capacity and social character (A). The approach is forward-casting, in that we use H and A descriptors to define strata because we assume they will be associated with health and development variability in children; they cannot be predictive at this stage. Our indices are similar to other descriptive indices such as the Human Development Index (HDI), a descriptor of development/poverty status that weights its three constituent variables (indicators of education, income, and health) equally [United Nations Development Programme (UNDP) 2009]. Two methods of combining H and A indices into a vulnerability rating (V) were explored. The first, V = H/A, seems logical, because vulnerability is inversely related to adaptation. However, V = 1/1 = 1 is not equivalent to V = 5/5 = 1, because one unit of H is not the same as one unit of A. (Contrast this with the well-known economic metric of cost/benefit ratio). The preferred method preserved information in the form V = [H,A], ranging from 5,1 (worst) to 1,5 (best), yielding 25 possible combinations (Table 1).

### Hazards/stressors exposure index (H)

Three attributes/variables/indicators were used to derive H. Population stress was classified based on population density (people per square mile per block-group in 2000) (MassGIS 2009). Road/transportation stress—a proxy indicator of air pollution, traffic accident risk, built-environment stressors such as traffic congestion, and the urban–rural continuum—was classified based on average daily traffic density [estimated average daily numbers of cars per square mile per block-group, obtained from Massachusetts Executive Office of Transportation (MassDOT 2009)]. Pollution stress was classified according to the density of stationary sources of pollution including 21E Massachusetts Contingency Plan waste cleanup sites, Toxics Release Inventory (TRI) toxics release sites, and Superfund sites [specifically, the total number of stationary pollution sources per square mile per block-group based on TRI and superfund site locations downloaded from the U.S. Environmental Protection Agency (U.S. EPA 2009) and state waste cleanup sites obtained from MassGIS (2009)]. In each case, a higher indicator value indicated higher stress.

Individual indicator values were aggregated to population-weighted average values for their parent town and standardized to a range of values between 0 and 10:


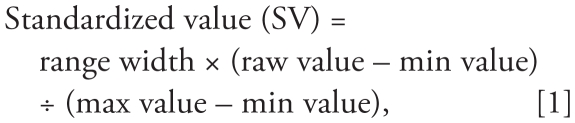


where range width is the difference between maximum and minimum standardized values (in our case, range width = 10), raw value is the value of an indicator for a town, and min and max values are the minimum and maximum values for this indicator in the entire data set.

Once standardized, variables were aggregated into a composite index H_s_ (scale 0–10) using multicriteria tools in geographic information systems (GIS) (Eastman 1999):


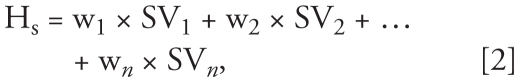


where w_1_ – w*_n_* are weights assigned respectively to *n* variables/indicators, and SV_1_ – SV*_n_* are standardized values of these indicators for each town.

Values of H_s_ were then divided into five classes of H (with cut points determined by natural breaks in data values), using classification tools in ArcGIS software (ESRI, Redlands, CA): very low (1), low (2), moderate (3), high (4), and very high (5).

### Adaptive capacity/social character index (A)

The A index comprised five variables from the U.S. Census 2000 (U.S. Census 2001). Education level was classified based on the percentage of the population > 25 years of age with high school as the highest level of education. Economic level was based on two indicators: income (median annual household income) and poverty level (percentage of households at or below the poverty level). Linguistic isolation level was determined by the percentage of households that were not primarily English-speaking. Minority level was determined by the percentage of the population from minority groups (e.g., black, Latino, Asian). Higher values of education level, poverty level, and linguistic isolation level indicate lower adaptive capacity, and higher income level indicates higher adaptive capacity. Although it is not appropriate to infer that adaptive capacity is a function of race or ethnicity in isolation, a population with high proportions of minority residents in conjunction with very low adaptive capacity based on other indicators is considered a highly vulnerable social group: an “environmental justice” population. As with individual H indicators, block-group values of individual A indicators were aggregated to population-weighted average values at the town scale, then standardized and aggregated and divided into five classes so that the final index A ranges from 1 to 5.

**Figure 2:**
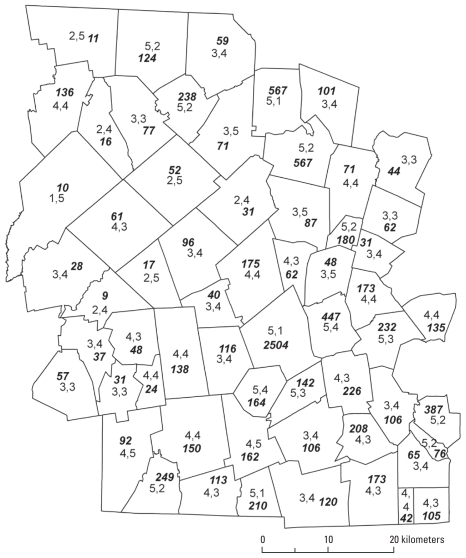
Map showing [H,A] ratings and MOS by town. Number in italics is MOS (expected births/year).

Figure 2 shows the vulnerability rating and MOS for each town. Towns range from very low hazard, very high adaptive capacity rating (1,5) for Petersham to very high hazard, very low adaptive capacity (5,1) for parts of Worcester City, a full spectrum of possible ratings.

**Figure 3:**
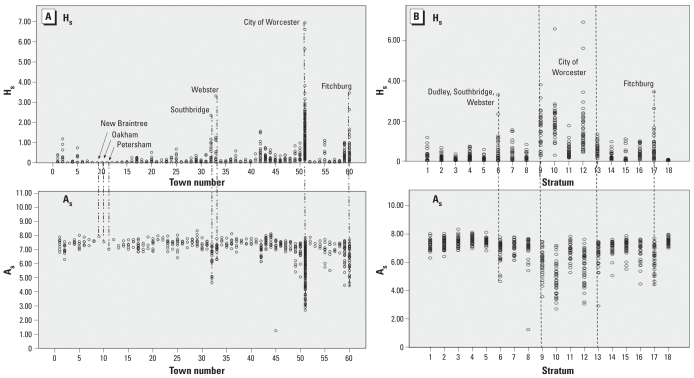
(*A*) Relative homogeneity within towns. Unclassified index values H_s_ (top) and A_s_ (bottom) are shown. Each dot is a constituent block-group value; small towns have only one block-group. (Final town values are a population-weighted average of the input block-group variables used to construct the indices.) (*B*) Relative homogeneity within strata. Each dot is a constituent block-group value of H_s_ or A_s_.

We sought to delineate 18 strata as a reasonable number to capture overall heterogeneity; each stratum was required to have an MOS within 10% of the county MOS divided by the number of strata (2,113 based on 2001–2008 data, giving an acceptable MOS range for each stratum of 1,902–2,324). Consistent with multivariable sampling stratification theory (Mulvey 1983), we attempted to achieve optimal relative homogeneity within each stratum. Figure 3A,B demonstrates this by showing how standardized unclassified values of H_s_ and A_s_ indices (scale 0–10) for constituent block-groups are closely grouped in towns and strata, respectively.

**Figure 4:**
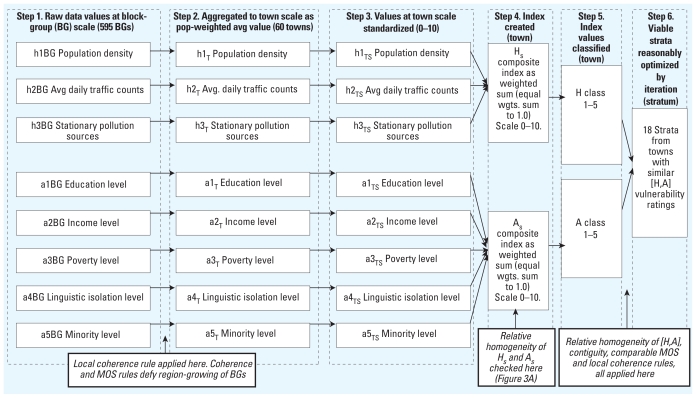
Flow chart of stratification approach. Six steps: input variables h1–h3, a1–a5; indices H, A; spatial scales; and rules used. Abbreviations: avg, average; pop, population; _T_ denotes town scale; _TS_ standardized town scale.

Final strata were composed of towns with comparable [H,A] ratings (except strata 1 and 15; see “Discussion”) and comparable MOS (Table 2). However, Worcester City was handled differently from the rest of the county because of its much higher expected births/year (MOS_City_ = 10,385; 2001–2008 data). Thus the city was divided into five strata (MOS_City_/average MOS = 10,385/2,113 = 4.91). In this case, sufficient contiguous relative homogeneity existed for the H and A indices calculated at block-group level to be used; thus, similar block-groups were aggregated into five strata. This left 13 strata to capture the rest of the county. Outside Worcester City, strata were created by combining contiguous towns using two criteria: *a*) similarity among towns in terms of vulnerability rating [H,A] (so the strata achieve reasonably optimal relative homogeneity), and *b*) aggregation obeys the MOS rule. To achieve a reasonably optimal result required several iterations on combinations of towns and summations of town MOS (Table 2). Some resulting strata are large in geographic size and consist of several towns. For example, we had to combine 12 sparsely populated contiguous towns for stratum 18 to achieve comparable MOS. In Table 2, strata are grouped to show comparable MOS, whereas data on H and A indices show relative homogeneity of the combination of the two [H,A] within strata, with the exception of two strata (1 and 15; see “Discussion”). Together with the requirement for contiguity of strata and conformity to local concepts of space, the data support the chosen configuration as a reasonably optimal multivariable stratification solution. Figure 4 shows the full stratification approach in six steps.

The final stage of stratification was to share the proposed map of viable strata with our community advisory board (CAB). The Mass CHILD CAB comprises representatives from subregions of the county and from social groups that include health and social agencies, local community leaders, and others with local knowledge. The consultation with CAB was important for three reasons: to check that the proposed strata conformed to local concepts of space and address any concerns that they were not coherent; to inform members about the method and sampling rules; and to engender a sense of joint ownership of the project among participants. CAB input led us to reconsider three strata because of concerns of some people over boundaries. We attempted to create alternative configurations, but each attempt violated the rule of comparable size, and we retained the original configuration. Although the CAB input did not result in any changes to proposed strata, it did offer the opportunity to explain boundaries and sampling constraints more clearly and the opportunity to work collaboratively on community member concerns. The first stratification proposal we sent to the NCS coordinating center for approval was rejected because it strayed too far from the comparable MOS requirement. We revised the proposal, shared it again with the CAB, and sent it to the coordinating center, which approved it.

### Segmentation

Once stratification was approved, the coordinating center divided each stratum into an equal number of segments, each of comparable MOS, and randomly selected one segment. Selected segments were checked for homogeneity using sociodemographic variables. The segmentation proposal was then sent to us for our approval; five strata had two segment options. Given the importance of involving community stakeholders in each stage of the project, we undertook an in-depth consultation with community representatives countywide over a period of 2 months. The purpose of the consultation was to inform local groups about the proposed segmentation, choose among any options, gain feedback on viability (revealing any fatal flaws, e.g., segments divided by physical barriers such as rivers or major highways), gain insights about recruitment strategy, and continue to engender joint ownership. It must be stressed that meaningful community consultation and involvement needs to happen well before recruitment plans are formulated so that local groups are fully informed and willing and prepared to collaborate. CAB members signed a confidentiality agreement so that we could enlist their ideas and concerns without making segments public.

## Results

**Figure 5:**
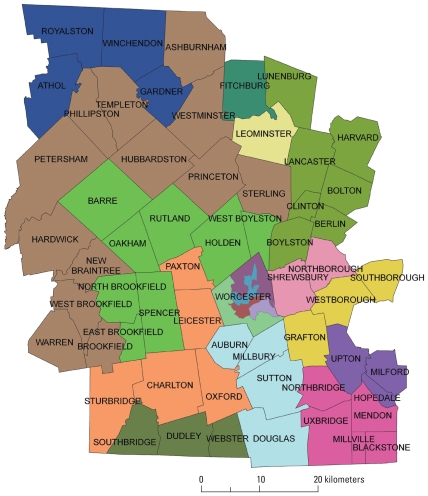
Final stratification map; shows 18 strata, 5 within the city of Worcester.

At the time of writing, 18 segments have been chosen randomly, one per stratum, and we are proceeding with a comprehensive characterization of each segment before recruitment begins. In each segment, we will recruit about 18 mothers/year for 4 years (1,000/18 = 55; 55/4 = 14; 14 + 30% ≈ 18 to account for loss and dropout). Figure 5 shows the final approved map of 18 strata, a reasonably optimal multivariable stratification solution.

## Discussion

### Data aggregation to strata

Spatial sampling for health research is an emerging field (Kumar 2007, 2009; Lee et al. 2006). Mulvey (1983) notes that the best stratification is not always required in terms of achieving the minimization of within-group variances and that good approximate solutions can be found without a significant loss of precision in sample estimates. This is relevant to our case, because we have three aspects that complicate pure univariable stratification: *a*) the use of eight variables in the form of two indices, so eight variances are in play; *b*) the two indices tend to be inversely correlated—when one is high, the other is low; *c*) the conditionality of equal MOS. Given these, we pursued the practical goal of achieving reasonably optimal relative homogeneity within strata and reasonably optimal relative heterogeneity between strata. Data in Table 2 and Figure 3A,B support this claim, but limitations need discussion. Although 16 strata are relatively homogeneous with comparable [H,A], two (1 and 15) are problematic (Table 2). Stratum 1 (Athol, Gardner, Royalson, Winchendon) lies in the top northwestern corner of the county, and stratum 15 (Berlin, Bolton, Boylston, Clinton, Harvard, Lancaster, Lunenburg) lies in the top northeastern corner. In both cases, after numerous iterations on combinations of towns (Figure 4, step 6) to meet the MOS, contiguity, and similarity requirements, the chosen solution (Figure 5) had to relax the similarity need in those strata, trading it off in optimization of the whole. It is worth noting that in both cases, the geographic location of these towns along the border of Worcester County reduces the number of potential town combinations for forming contiguous strata (compared with nonbordering towns).

### Limitations and alternative approaches

As with any model, there are strengths and weaknesses. Whereas the main strength lies in the information value of the stratification indices, the main limitation is that relative homogeneity within strata can be challenging. Our interactions with other NCS sites show that all are struggling in a productive way, as we did, with how to stratify. There are alternative approaches, and we considered them. Picking community-accepted boundaries up front, configuring areas for comparable MOS and then checking homogeneity afterward is an option. The Queens County Vanguard Site used community boundaries previously defined by the New York City Department of City Planning in 2007 to delineate 18 strata, then checked for homogeneity afterward using census data on race, ethnicity, education, income, and foreign-born status (Lioy et al. 2009). Another option is to pick only one or two key variables (perhaps one for hazard, one for adaptation) as the basis of stratification. This would simplify matters greatly in terms of achieving higher relative homogeneity within strata but would have less information value and may reduce our ability to detect associations among variables and health outcomes down the line. Using the smallest unit of analysis, the block, as our starting point was considered but abandoned because insufficient data are available at that scale. Even if we had data at this scale, the resulting spatial heterogeneity would have been much greater still than at the block-group scale, and aggregation would have been more difficult given MOS and local coherence rules. These rules also prevented the use of region-growing algorithms for aggregation (Brookes 1997). After weighing options, we chose the multivariable vulnerability index approach, because we wanted to capture known high heterogeneity in hazard indicators and adaptation indicators up front as the health-centered basis for stratification. The requirements of MOS, contiguity, and local coherence were secondary. However, as we discovered and have described herein, the tradeoff is that even a reasonably optimal solution is more elusive, because although heterogeneity between strata can be achieved, homogeneity within each is challenging given the sampling rules.

The jigsaw puzzle is complex: How do you capture health-relevant heterogeneity in a county using < 20 viable strata? In our case, the intermediate locally coherent spatial scale chosen was the town: 13 of the 18 strata were made up from 59 towns (the city of Worcester is one town with five strata), yielding an average of 4.5 towns per stratum. The town is not too big an intermediate scale, but would be too big a starting scale. In sum, we are confident that the solution is reasonably optimal for meeting our goals of heterogeneity between strata and homogeneity within strata. Discussions with our community advisors were fruitful in terms of validating the vulnerability ratings of towns and the coherence of the strata. Although not a sampling requirement per se, this approval is essential for a functional community–researcher partnership, one governing of future success.

The lack of a standardized NCS protocol for stratification of target populations by factoring in the diverse environments they inhabit is both a challenge and an opportunity. What potential does our approach have to inform stratification at other study sites? The method used extant data and offers potential to inform stratification at other sites by remaining adaptable to local conditions and data availability. Several important results from our study are relevant to the other study sites. In theory, the two components of our vulnerability approach, a proxy of hazards/stressors exposure and a measure of social adaptation/demographic character, could be tailored to each study site according to its own local conditions and data availability. The importance of spatial perspective and GIS expertise in our study center became obvious early on in the process. Even though most of our index calculations were done using spreadsheet operations, GIS allowed us to visualize input and output data and analyze their spatial characteristics. It became an important focus for our analytic efforts and played a key role in our CAB discussions.

In practice, more general applicability of our GIS-enabled approach is limited by three factors: particular differences in the extent of technical focus on GIS among sites; availability of relevant data; and variability in geographic and demographic organization among sites. Based on discussions with other NCS centers and the coordinating center, we judge enabling GIS to have moderate potential *in situ* at present, but moderate to high in terms of building future capacity. U.S. Census data are rich sources of sociodemographic and economic data that could serve as the basis for spatial stratification, and we strongly recommend NCS Study Centers take full advantage of them. Similarly, pollution sites data collected by the U.S. EPA and the states (TRI, Superfund sites, and state-level hazardous waste sites) are useful sources. All of our indicators, except for average daily traffic count, come from publicly available national databases; we judge data availability for the same or similar indicators to be high. For variability in geodemographic organization, we chose a random sample of 10 wave 2–wave 3 sites (2008–2010 locations) (NCS 2010). Table 3 summarizes site characteristics and similarities with Worcester County and assesses the potential of our method to inform each site. The average rating is moderate. Combining ratings for the three criteria, we judge our approach has moderate to high potential to inform sampling at other sites.

### Opportunities for improved technical exchanges between sites using GIS

The NCS offers an opportunity, building on and developing the resources at > 100 sites, to help initiate a program of environmental health mapping using different types of public domain data. Such an effort could create a network of health-GIS resources nationwide that would have as its primary purpose the support of NCS objectives and better communication and coordination between the NCS sites. GIS technology, because it combines possibilities for visualization with sophisticated tools for analysis, is well suited as a vehicle for communication and coordination. The creation of such a network will provide opportunities for further network database development to serve multiple public health and environmental objectives.

### Community engagement

Adequate community consultation and involvement needs to happen during each stage of the project: stratification, segmentation, characterization, recruitment, data gathering, and follow-up. For example, unless consultation happens before recruitment plans are formulated, the project runs the risk of pushback and a feeling that the work is imposed on local communities, hospitals, and residents. We have found that GIS capabilities facilitate community engagement because they provide the opportunity on the spot to create maps with multiple features in response to stakeholder concerns. The need for meaningful engagement extends to participants: without a sense of joint ownership of the research and tangible benefits that improve health over the 21-year period, retention will suffer.

## Conclusion

Multivariable, health-relevant stratification up front may improve detection power for environment–child health associations down the line. Our method used extant data and offers noteworthy potential to inform stratification at other sites. The approach is adaptable to local conditions and data availability, and the GIS focus should facilitate data comparisons between sites. The approach with its GIS manifestation also encouraged meaningful community engagement, which we consider likely to promote sustainability of the Worcester County NCS effort. Some key capacity building in the use of environmental health science–GIS for communication between NCS sites would be desirable. We see exciting opportunities to pursue new analytic methods development and to craft meaningful collaborations among researchers, communities, and participants.

## Figures and Tables

**Table 1:** Vulnerability rating scheme V = [H,A] using five classes for both indices.

Hazard index (H)	Adaptation index (A)				
	Very low	Low	Moderate	High	Very high
Very low	1,1	1,2	1,3	1,4	1,5 (best)
Low	2,1	2,2	2,3	2,4	2,5
Moderate	3,1	3,2	3,3	3,4	3,5
High	4,1	4,2	4,3	4,4	4,5
Very high	5,1 (worst)	5,2	5,3	5,4	5,5

**Table 2:** Data summary by stratum and town.

							V rating [H,A]										
S no.	Town name	Town no.	Town pop	S pop	H_s_ town	A_s_ town	H town	A town	MOS	S MOS^a^	No. BGs	Min_BG pop	Max_BG pop	M_BG H_s_	SD_BG H_s_	M_BG A_s_	SD_BG A_s_
1	Athol	1	11,299		0.936	6.46	4	4	531		8	1,039	2,092	0.150	0.155	7.40	0.364

1	Gardner	2	20,770		2.265	4.63	5	2	917		12	706	2,512	0.330	0.375	7.04	0.340

1	Royalston	3	1,254		0.005	7.11	2	5	41		1	1,254	1,254	0.005	0.000	7.11	0.000

1	Winchendon	5	9,611	42,934	2.007	4.24	5	2	479	1,969	7	643	2,230	0.250	0.264	7.39	0.341

2	Barre	16	5,113		0.730	5.55	4	3	232		4	804	1,595	0.090	0.113	7.50	0.239

2	E. Brookfield	22	2,097		0.543	6.33	4	4	80		2	687	1,410	0.080	0.092	7.76	0.127

2	Holden	17	15,621		0.868	6.21	4	4	670		13	667	1,978	0.140	0.090	7.25	0.279

2	N. Brookfield	24	4,683		0.965	5.39	4	3	176		5	661	1,380	0.140	0.126	7.40	0.224

2	Oakham	10	1,673		0.030	7.49	2	5	77		1	1,673	1,673	0.030	0.000	7.49	0.000

2	Rutland	19	6,353		0.271	6.26	3	4	339		3	1,329	2,819	0.040	0.036	7.43	0.422

2	Spencer	25	11,691		1.338	6.15	4	4	513		10	637	1,870	0.200	0.226	7.37	0.490

2	W. Boylston	20	7,481	54,712	1.210	5.72	4	3	224	2,310	8	547	2,509	0.170	0.162	7.34	0.342

3	Charlton	28	11,263		0.659	6.68	4	4	591		6	527	2,925	0.080	0.083	7.56	0.166

3	Leicester	23	10,471		0.470	6.57	3	4	429		8	914	2,410	0.070	0.043	7.43	0.344

3	Oxford	29	13,352		1.123	7.48	4	5	580		9	565	2,882	0.150	0.108	7.68	0.349

3	Paxton	18	4,386		0.499	6.30	3	4	148		4	614	1,492	0.070	0.062	7.16	0.274

3	Sturbridge	30	7,837	47,309	0.550	7.76	4	5	353	2,101	6	1,054	1,702	0.070	0.046	7.30	0.311

4	Auburn	43	15,901		2.238	6.29	5	4	612		20	500	1,379	0.300	0.207	7.56	0.259

4	Douglas	34	7,045		0.240	6.81	3	4	494		4	798	2,319	0.040	0.043	7.62	0.215

4	Millbury	44	12,784		1.884	5.53	5	3	539		13	573	1,523	0.240	0.186	7.66	0.340

4	Sutton	35	8,250	43,980	0.315	6.27	3	4	391	2,036	6	624	3,090	0.040	0.036	7.40	0.224

5	Blackstone	37	8,804		1.335	5.82	4	3	418		6	749	3,372	0.240	0.207	7.39	0.363

5	Mendon	38	5,286		0.347	6.84	3	4	224		3	1,044	2,640	0.050	0.021	7.58	0.106

5	Millville	39	2,724		0.590	6.57	4	4	177		3	607	1,187	0.100	0.067	7.53	0.201

5	Northbridge	46	13,182		0.717	5.72	4	3	757		9	465	2,934	0.110	0.092	7.42	0.112

5	Uxbridge	36	11,156	41,152	0.682	6.02	4	3	625	2,202	7	654	2,610	0.090	0.065	7.54	0.328

6	Dudley	31	10,036		0.957	5.85	4	3	465		7	877	2,043	0.140	0.171	7.38	0.194

6	Southbridge	32	17,214		3.581	4.98	5	2	971		17	556	1,833	0.530	0.595	6.43	0.829

6	Webster	33	16,415	43,665	5.602	2.42	5	1	840	2,275	13	525	2,754	0.680	0.907	7.06	0.403

7	Hopedale	41	5,907		1.967	4.78	5	2	304		3	1,524	2,709	0.240	0.174	7.60	0.137

7	Milford	42	26,799		4.183	4.27	5	2	1,450		19	663	2,863	0.610	0.482	6.89	0.428

7	Upton	40	5,642	38,348	0.312	6.68	3	4	330	2,084	4	644	1,812	0.060	0.042	7.55	0.183

8	Grafton	45	14,894		1.062	5.97	4	3	909		10	324	4,332	0.140	0.113	6.76	1.951

8	Southborough	48	8,781		1.091	6.44	4	4	415		6	796	2,742	0.140	0.079	7.32	0.257

8	Westborough	49	17,997	41,672	2.162	5.74	5	3	834	2,158	12	616	3,100	0.280	0.247	6.70	0.667

9	Worcester	51	NA	29,690	NA	NA	4	3	2,075	2,075	29	488	2,367	1.599	0.869	5.98	0.954

10	Worcester	51	NA	32,800	NA	NA	5	2	2,155	2,155	36	415	1,743	1.970	0.982	4.84	1.144

11	Worcester	51	NA	37,984	NA	NA	3	4	2,041	2,041	30	424	3,305	0.526	0.355	6.78	0.618

12	Worcester	51	NA	35,627	NA	NA	4	3	2,096	2,096	40	514	2,123	1.652	1.311	5.69	1.145

13	Worcester	51	NA	36,547	NA	NA	4	4	2,018	2,018	32	428	3,538	0.592	0.311	6.63	0.919

14	Northborough	47	14,013		1.384	6.58	4	4	607		9	787	2,766	0.190	0.124	7.25	0.259

14	Shrewsbury	50	31,640	45,653	2.349	6.57	5	4	1,698	2,305	19	543	3,150	0.330	0.238	6.90	0.588

15	Berlin	52	2,380		0.353	6.80	3	4	91		3	394	1,294	0.040	0.017	7.63	0.236

15	Bolton	53	4,148		0.260	5.76	3	3	245		2	1,831	2,317	0.040	0.007	7.33	0.156

15	Boylston	54	4,008		0.423	7.20	3	5	175		4	603	1,787	0.070	0.066	7.34	0.220

15	Clinton	55	13,435		3.659	3.93	5	2	739		9	856	3,904	0.540	0.393	6.89	0.633

15	Harvard	56	5,981		0.264	5.91	3	3	156		6	734	1,202	0.040	0.019	6.89	0.748

15	Lancaster	57	7,380		0.577	6.81	4	4	251		4	720	2,966	0.080	0.064	7.06	0.245

15	Lunenburg	58	9,401	46,733	0.373	6.18	3	4	353	2,010	6	1,109	2,208	0.060	0.033	7.26	0.260

16	Leominster	59	41,303	41,303	3.115	4.53	5	2	2,037	2,037	25	608	3,330	0.470	0.297	6.74	0.791

17	Fitchburg	60	39,102	39,102	5.021	2.54	5	1	2,181	2,181	34	104	2,207	0.720	0.732	6.40	0.849

18	Ashburnham	6	5,546		0.159	6.11	3	4	254		5	788	1,843	0.030	0.021	7.53	0.240

18	Brookfield	21	3,051		0.234	5.86	3	3	125		3	929	1,162	0.030	0.027	7.79	0.162

18	Hardwick	7	2,622		0.385	6.59	3	4	100		2	900	1,722	0.050	0.064	7.50	0.297

18	Hubbardston	8	3,909		0.060	7.00	2	5	198		2	1,310	2,599	0.010	0.000	7.61	0.021

18	New Braintree	9	927		0.015	7.94	2	4	39		1	927	927	0.015	0.000	7.94	0.000

18	Petersham	11	1,180		0.000	7.02	1	5	37		1	1,180	1,180	0.000	0.000	7.02	0.000

18	Phillipston	12	1,621		0.035	7.71	2	4	62		1	1,621	1,621	0.035	0.000	7.71	0.000

18	Princeton	13	3,353		0.055	6.17	2	4	102		2	1,371	1,982	0.010	0.007	7.21	0.120

18	Sterling	14	7,257		0.182	6.96	3	5	319		3	1,420	3,092	0.030	0.010	7.41	0.189

18	Templeton	4	6,799		0.297	5.30	3	3	255		6	502	1,930	0.040	0.025	7.51	0.123

18	Warren	26	4,776		0.257	5.32	3	3	96		4	1,025	1,450	0.040	0.013	7.50	0.090

18	W. Brookfield	27	3,804		0.346	6.80	3	4	138		3	1,114	1,567	0.050	0.045	7.48	0.323

18	Westminster	15	6,907	51,752	0.245	6.93	3	5	263	1,988	5	741	1,789	0.030	0.016	7.33	0.251

Abbreviations: A town, A classification for town; A_s_, standardized adaptation index value for town; H town, H classification for town; H_s_, standardized hazard index value for town; M_BG A_s_, mean block-group adaptation index; M_BG H_s_, mean block-group hazard index; Max_BG Pop – maximum block-group population; Min BG pop, minimum block-group population (2000 data); MOS, town measurement of size (no. expected births/year based on 2001–2008 data); NA, not applicable; No. BGs, number of block-groups; S MOS, stratum MOS; S no., stratum number; S pop, stratum population; SD_BG A_s_, standard deviation block-group adaptation index; SD_BG H_s_, standard deviation block-group hazard index; Town no., number of town in Figure 3A,B; Town pop, town population [as of 2000 (U.S. Census 2000)]; V rating [H,A], vulnerability rating. Strata are grouped to show comparable MOS (allowable range = mean ± 10%: 1,902–2,324 births/year). Data on H_s_ and A_s_ indices show relative homogeneity of the indices within towns and strata. Town ratings [H,A] within strata are relatively homogeneous except for strata 1 and 15, where a tradeoff was made for optimization of the whole (see “Limitations”). The H_s_, A_s_ values for towns are different from the average block-group values because we used a population-weighted approach, and population varies considerably by block-group as shown. Five towns (New Braintree, Oakham, Petersham, Phillipston, Royalston) have only one block-group per town, so their H_s_ and A_s_ values are the same as the average values].

a Mean ± SD = 2,113 ± 106.

**Table 3:** Site comparisons for information potential: random sample of 10 wave 2-wave 3 sites.

County	State	Population (2000)	Area, square miles	Geodemographic character	Similarity^a^— information potential
Worcester	MA	751,000	1,579	Mainly rural, wooded and small farms. Largest city Worcester (176,000 in 2006). Sixty incorporated cities/towns.	—
Benton	AR	153,000	846	Gentle rolling hills. Largest city Rogers (43,000 in 2003). Consists of 17 incorporated cities/towns, 6 unincorporated.	3
Humboldt	CA	127,000	4,052	Rural, densely forested. Seven incorporated towns. Largest cities Eureka and Arcada have 35% population; 13% population in other five towns; 52% in unincorporated communities.	2
Litchfield	CT	182,000	945	Rural. Occupies portion of Appalachian Mountains. Twenty-six cities/towns. Lowest population density in CT.	4
Polk	IA	374,000	592	Mainly urban, several lakes and rivers. Twenty cities, largest Des Moines (197,000 in 2008 est.).	3
Orleans (parish)	LA	484,000		Urban/major city with surrounding much smaller towns. Twenty-three towns, one incorporated. Largest city New Orleans; > 30 other populated villages/communities.	2
Stearns	MN	133,000	1,390	Rolling hills, scenic lakes. Thirty-two cities, 34 townships.^b^ Largest city St. Cloud (59,000 in 2000).	3
Coahoma	MS	31,000	583	In Mississippi Delta region. Largest city Clarksdale (21,000 in 2000). Six incorporated towns, nine unincorporated communities.	3
Cumberland	NC	303,000	658	Coastal plain topography. Eleven townships.^b^ Largest city Fayetteville (121,000 in 2000).	3
Burlington	NJ	423,000	819	Coastal and alluvial plain. Forty cities/towns/townships. Largest city Evesham (42,000 in 2000).	3
Lamar	TX	48,000	932	Rolling hills, open spaces. Sixteen cities/towns, seven incorporated. Largest city Paris (26,000 in 2000).	3
Average					3

a To Worcester, MA. Rating 1, 2, 3, 4, 5: very low, low, moderate, high, very high.

b Small area with local government.
